# An Approach toward Artificial Intelligence Alzheimer’s Disease Diagnosis Using Brain Signals

**DOI:** 10.3390/diagnostics13030477

**Published:** 2023-01-28

**Authors:** Seyed-Ali Sadegh-Zadeh, Elham Fakhri, Mahboobe Bahrami, Elnaz Bagheri, Razieh Khamsehashari, Maryam Noroozian, Amir M. Hajiyavand

**Affiliations:** 1 Department of Computing, School of Digital, Technologies and Arts, Staffordshire University, Stoke-on-Trent ST4 2DE, UK; 2 Behavioral Sciences Research Center, School of Medicine, Isfahan University of Medical Sciences, Isfahan 8174533871, Iran; 3 Quality and Usability, Technical University of Berlin, 10623 Berlin, Germany; 4 Cognitive Neurology and Neuropsychiatry Division, Department of Psychiatry, Tehran University of Medical Sciences, Tehran 1416634793, Iran; 5 Department of Mechanical Engineering, School of Engineering, University of Birmingham, Birmingham B15 2SQ, UK

**Keywords:** Alzheimer’s disease, diagnosis, electroencephalogram (EEG), machine learning, data augmentation strategy

## Abstract

Background: Electroencephalography (EEG) signal analysis is a rapid, low-cost, and practical method for diagnosing the early stages of dementia, including mild cognitive impairment (MCI) and Alzheimer’s disease (AD). The extraction of appropriate biomarkers to assess a subject’s cognitive impairment has attracted a lot of attention in recent years. The aberrant progression of AD leads to cortical detachment. Due to the interaction of several brain areas, these disconnections may show up as abnormalities in functional connectivity and complicated behaviors. Methods: This work suggests a novel method for differentiating between AD, MCI, and HC in two-class and three-class classifications based on EEG signals. To solve the class imbalance, we employ EEG data augmentation techniques, such as repeating minority classes using variational autoencoders (VAEs), as well as traditional noise-addition methods and hybrid approaches. The power spectrum density (PSD) and temporal data employed in this study’s feature extraction from EEG signals were combined, and a support vector machine (SVM) classifier was used to distinguish between three categories of problems. Results: Insufficient data and unbalanced datasets are two common problems in AD datasets. This study has shown that it is possible to generate comparable data using noise addition and VAE, train the model using these data, and, to some extent, overcome the aforementioned issues with an increase in classification accuracy of 2 to 7%. Conclusion: In this work, using EEG data, we were able to successfully detect three classes: AD, MCI, and HC. In comparison to the pre-augmentation stage, the accuracy gained in the classification of the three classes increased by 3% when the VAE model added additional data. As a result, it is clear how useful EEG data augmentation methods are for classes with smaller sample numbers.

## 1. Introduction

AD is a degenerative neurological disorder characterized by the slowing of background oscillations or the downward shift of fundamental brain oscillations [1]. This is the first cause of dementia, and the major sign is fast cognitive deterioration. It was estimated that 35.6 million people lived with dementia worldwide in 2010, with numbers expected to almost double every 20 years, to 65.7 million in 2030 and 115.4 million in 2050. In 2010, 58% of all people with dementia lived in countries with low or middle incomes, with this proportion anticipated to rise to 63% in 2030 and 71% in 2050 [2]. Cortical neuron loss, axonal disease, and cholinergic deficits are hypothesized to cause these disorders [3,4]. The condition is more common in those over the age of 65, and the rate of occurrence rises exponentially with age [5]. The number of people with neurological disorders is about 50 million and is projected to exceed 100 million by 2050 [6]. An intermediary stage between Healthy Ageing (HA), sometimes referred to as Healthy Control (HC), and AD has been widely recognized as a stage called MCI. MCI is a term that refers to persons who have many Alzheimer’s disease symptoms but not enough to fulfil the diagnostic criteria for the disease and with more cognitive loss than is typical for their age [7]. Patients with MCI may gradually regain normalcy. Recent studies estimated that the conversion rate from MCI to AD is approximately 15% per year [4], whereas this rate is only 1–2% of the global population [8]. While there is no known cure for AD or MCI, diagnosis and treatment of them in the early stages of the condition can considerably slow the disease’s course and delay the progression of MCI to dementia. As a result, early detection of AD and MCI is critical [9,10].

In ref. [10] after performing pre-processing and dividing the signal into 5-s windows, extracting latent features from 2D gray-scale representations of PSD spectra, and then using a convolutional deep learning network to separate the three classes, AD vs. MCI vs. HC, an accuracy of 83.33% was achieved. Alzheimer’s disease and other neurological disorders such as epilepsy, stroke, and Parkinson’s disease can be diagnosed using a variety of methods. These include Magnetic Resonance Imaging (MRI), Single Photon Emission Computed Tomography (SPECT), and Positron Emission Tomography (PET) [11]. Neuroimaging technologies that are inexpensive and readily available, such as EEG, have also been examined for the diagnosis of AD. Electroencephalography (EEG) is a non-invasive, low-cost, and portable technique, with a high temporal resolution, that reflects the electrical activity of the brain [12]. EEG is a reliable tool for the early detection of AD, according to a number of clinical investigations, because the disease has an effect on the complexity, synchronization, and rhythm of the signals, which reduces complexity and causes rhythm to slow. The slowing of the rhythm in AD patients’ EEG signals can be explained by an increase in theta and delta frequency range activity and a decrease in alpha and beta frequency range activity [13]. Differentiating Alzheimer’s disease from MCI and healthy people has been a challenging issue in research as well. For this reason, the effect of various methods such as MRI, FMRI, EEG, etc. in differentiating these three cases has been studied. In the meta-analysis study that investigated olfactory function to differentiate these three cases, it was shown that olfactory identification was more profoundly impaired in patients with AD than in those with MCI [13]. In another study, using EEG findings, an algorithm was designed to distinguish Alzheimer’s from MCI, and as a result, an accuracy, sensitivity, and specificity of 96.5%, 96.21%, and 97.96% were achieved [14].

Recently, novel DA methods have attracted the use of DNNs to map data space from high-dimensional to low-dimensional and realize feature extraction to reconstruct the artificial data. There are two typical deep learning strategies for DA: autoencoder (AE) and generative adversarial networks [14]. Most recent studies have employed binary classification to distinguish AD and MCI from HC in EEG data. An efficient approach for detecting AD using the EEG data of AD and HC patients was developed by [13]. They used three different feature sets and an SVM classifier (spectral-, wavelet-, and complexity-based features). Support vector machines (SVMs) are supervised learning models that examine data for regression and classification. They also include associated learning methods. As a consequence, they are able to attain a binary classification accuracy of 96% [12] performed a time-frequency analysis of EEG signals using Fourier transforms and wavelet transforms. Although they used EEG signals of the AD, MCI, and HC subjects, they performed only binary classification and obtained a maximum accuracy of 92% in dealing with HC and MCI. Few studies have compared AD, MCI, and HC [14] extracted statistical and spectral features from EEG data for the classification of AD, MCI, and HC. Using random forest classification, they were able to reach an accuracy of 88.79%. Subsequently [15] proposed a new method to discriminate between AD, MCI, and HC classes using time-frequency domain analysis with continuous wavelet transform (CWT) and bispectral representation (BiS). They fed the extracted features into a multilayer perceptron classifier and obtained an accuracy of 89.24% for a three-class scheme.

Data augmentation (DA) comprises the generation of new samples to augment an existing dataset by transforming the existing samples in a manner that increases the accuracy and stability of the classification or regression. Exposing the classifier to more variable representations of its training samples makes the model more invariant and robust to transformations of the type that it is likely to encounter when attempting to generalize to unseen samples. In recent years, DA techniques have received widespread attention and achieved appreciable performance boosts for DL on EEG signals [15]. The most important methods for EEG data augmentation include a noise addition (17%) deep learning method. We have two main categories for adding noise to the EEG signals for the purpose of DA: (1) Add various types of noise such as Gaussian, Poisson, salt-and-pepper noise, etc., with different parameters (for instance: mean and standard deviation to the raw signal; (2) Convert EEG signals to sequences of images and add noise to the images. In this paper, adding noise to the raw signal is used.

This study proposes a new approach based on EEG signals for discriminating between AD, MCI, and HC in two-class and three-class classifications. This method is based on PSD features and the SVM classifier. In addition, the dataset in this study has a class imbalance problem (CIP), which is a severely imbalanced distribution of classes. In the case of machine learning, the majority class overwhelms the minority class. The majority class gradient component is substantially longer than the minority class. As a result, the majority class dominates weight upgrades more than the other classes, resulting in a rapid decrease in the majority class error and an increase in the minority class error. Oversampling, down-sampling, and algorithmic-level methods such as the threshold-moving technique and cost-sensitive learning are among the most common CIP handling methods. This research focuses on data augmentation strategies [16], which are a type of excessive sampling methodology. Data augmentation is the process of creating new samples to supplement current data sets and improve classification or regression accuracy and stability [17]. It usually generates additional samples from lesser-known classes to match the number of samples in each class, resulting in new data collection. One of the major challenges for machine-learning algorithms that operate under the assumption that data is evenly distributed across classes is class imbalance in imbalanced AD data. For the first time in the AD domain, different approaches to data augmentation—noise addition [16], VAE-based data production [14], and hybrid—are used independently and evaluated in this study, which is based on classic methods and deep neural networks.

The following sections of the paper are organized as follows. Section 2 describes the studied dataset and explains the signal pre-processing, feature extraction, and classification methods used in this paper. Section 3 displays the results of the study, and Section 4 contains the discussion. Finally, Section 5 presents the conclusions and further work.

## 2. Experiments

### 2.1. Dataset Description

We conducted experiments on the [18], which contains the EEG data of 59 patients with moderate AD (MMSE score 19-19) and 7 patients with MCI. Healthcare practitioners administered the mini-mental state examination (MMSE) to people who could have dementia. Short- and long-term memory, concentration, and understanding of instructions are all assessed using the MMSE. A clinical history was taken from the patient and the patient’s caregiver. At the time of diagnosis, the general practitioner asked for information about comorbidity, and all patients underwent neurological and physical testing. The HC group included 102 adults with no memory or cognitive impairment and no other neurological illnesses. The AD group was 70.5 ± 4.9 years old, the MCI group was 67 ± 7.67 years old, and the HC group was 72.2 ± 5.3 years old. The AD group had 28 men and 31 women, the MCI group had 3 men and 4 women, and the HC group had 43 men and 59 women. The same standard conditions are used for all EEG recordings. Participants sat comfortably on the bed and closed their eyes. The same standard conditions were used for all EEG recordings. Nineteen EEG electrodes were placed on the scalp following the 10–20 system. Using a Medelec Valor digital amplifier with a sampling rate of 256 Hz, each participant was instructed to maintain complete stillness and keep their eyes closed while their EEG was being recorded.

### 2.2. Pre-Processing of EEG Signals

EEG signals are susceptible to noise, which might make weaker EEG signals incomprehensible. Blinking or moving muscles are examples of artefacts that can influence data and alter EEG signals. Pre-processing is usually referred to as the reduction in background noise from the data in order to approach brain signals in the EEG data. There is no single standard for preprocessing EEG signals because this is still an active area of research. This means that researchers have complete control over how raw data is modified.

In this study, using the EEGLAB toolbox, various pre-processing techniques were applied to EEG data. The locations of 19 primary channels, including FP1, FP2, F7, F3, Fz, F4, T7, C3, C4, T8, P7, P3, P4, P8, O1, and O2, were first identified by loading raw data into EEGLAB. The 128 Hz sample rate was then selected to equalize the sampling rate of all the data, and a band-pass filter was used to select frequencies between 1 and 50 Hz [8,10]. In the next step, the data was reviewed, and the channels that generated ruined signals were considered inappropriate channels and eliminated using the artifact subspace reconstruction (ASR) plugin. After that, the interpolation process was used to equalize the number of channels in all of the data. The ASR plugin was also used to remove artefacts recorded during samplings, such as blinking and muscle movements. Finally, the data was divided into a number of independent components using the independent component analysis (ICA) tool, and each of these components was checked and labelled using the ICLabel plugin. In the end, any component that was not labelled “brain” was eliminated from the data.

### 2.3. Method

AE is a feed-forward neural network used to encode the raw data into low-dimensional vector representations by one-half of the network and to reconstruct these vectors back into artificial data using another half of the network. To obtain the expected generated data, a variational autoencoder (VAE) was proposed to improve the performance of the autoencoder. Compared with AE, a VAE ensured that generated data was subject to specific probability distribution by adding constraints into the structure [14].

We first give a quick rundown of the autoencoder to help comprehend the process better. The encoder, the decoder, and the latent space are the three primary parts of the autoencoder. The encoder uses neural networks to compress input data. The aim is to maximize the distribution of data in the latent space, which is where all the information is stored. In contrast, the decoder works to reconstruct the input data from the compressed latent space, making it a mirror of the encoder. The autoencoder’s ultimate objective is to deliver a dimensionality reduction algorithm that gains encoding knowledge by minimizing a distance metric loss.

After pre-processing, the data was divided into specified intervals. The study included 5, 10, and 15 s as essential time intervals. The average PSD, variance, mean, and zero-crossing rate are some of the variables that are employed by SVM to classify the data [19,20]. EEG signals recorded from Alzheimer’s patients reveal an increase in power at low frequencies, such as the delta and theta frequency bands, and a decrease in power at higher frequencies, such as the alpha, beta, and gamma frequency bands, according to research [21]. Therefore, PSD in various frequency bands is one of the approaches for distinguishing and classifying healthy people from those with AD and MCI. The PSD represents the power distribution of the signal’s frequency components. Mean PSD was the method used in this study to extract the aspects of the EEG signals. The average PSD in a frequency band corresponded to the average signal power in that band.

The signal was first filtered in the relevant frequency ranges, the PSD was then determined using the Welch function, and finally, the average was used to calculate this feature. Given that the dataset used in this study had 19 channels and that the number of frequency bands encompassing delta, theta, alpha, beta, and gamma was equal to 5, each person had 95 features. A total of 152 attributes were employed in the classification using SVM, taking into account the features of variance, mean, and zero-crossing rate (ZCR). The ZCR measures how quickly signal shifts from positive to zero to negative or from negative to positive. These traits are included in the table below (see Table 1).

**Figure 1:**
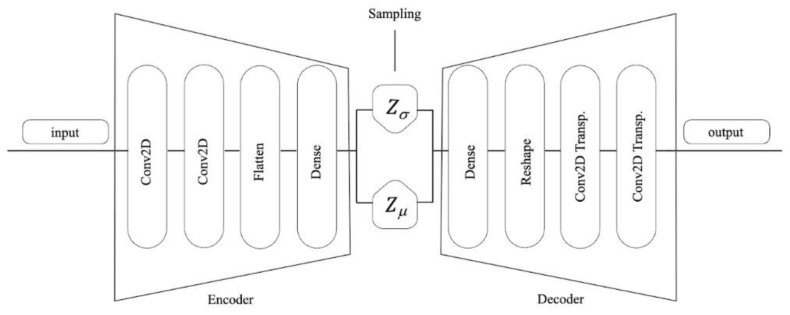
The diagram of the VAE.

Since there were fewer samples related to MCI patients than samples linked to healthy and AD patients, the dataset used in this study was imbalanced. Adding more MCI samples and using them in the model’s classification training will solve this problem. The data augmentation strategy included the use of noisy data, VAEs, and combinations of these two. The VAE was first trained using a dataset of inner speech [22] and the weights produced were taken as a starting point. Once the model had learned the distribution of the data, it was given data from the target data set, which consisted of MCI samples. As a result, data was generated at various intervals of 5, 10, and 15 s. The SVM model was used to train on this data at different time intervals. As shown in Figure 1, two convolution layers were placed before and after the sample layer in the architecture of the VAEs, and after obtaining channel information, different distributions were employed to produce random data in the center layer. The information was decoded, and the desired data was produced by the decoder network, which had a symmetric architecture to the encoder network.

#### Augmentation Techniques

Three augmentation techniques were used, namely VAE, noise addition, and a hybrid approach. Data augmentation was then carried out on the training set once the EEG data had been divided into the train, validation, and test sets. The split ratio for the train, validation, and test sets was 70:12:18. Data for each subject and class were grouped for all data creation techniques. For procedures that called for the direct synthesis of genuine trials, artificial trials were generated on a per-class basis.

##### Variational Autoencoder

The distribution of the data was learned, and simulated trials were generated based on actual trials using a conditional variational autoencoder. Similar to an autoencoder, the VAE comprises an encoder and a decoder. The encoder learns a representation of the data and encodes it by providing a lower-dimensional representation in the conventional encoder–decoder combination of an autoencoder. In contrast, the decoder takes the encoded representation and attempts to rebuild the signal in order to decode the representation. An autoencoder using a VAE does not just train a function that converts input into a compressed form and back to its original form. Instead, it gains knowledge of the probability distribution’s parameters. As a result, the learnt representation is limited by the data’s mean and standard deviation. By combining the labels with the trials during the learning process, we conditioned the learning. Convolutional layers comprised the architecture of the VAE. Oversampling the VAE’s initial training set produced better losses and training stability. The VAE learning process can be seen in Figure 1. Given its comprehensive approach to studying the distribution of real data before generating synthetic ones, the VAE differs from previous approaches.

As mentioned, VAE makes artificial EEG data, and a 2D convolutional autoencoder can be used (see Figure 1). Here, the encoder compressed the input data, while the decoder recovered the data’s key properties. We imported the data first, then set the model and data configuration parameters. The training data determined the configuration width and height settings. The data needed to be reshaped and the configuration settings applied before the encoder could be built. Three steps made up this procedure: We defined it first. In order to link the encoder and the decoder afterwards and instantiate the VAE as a whole, we also conducted what is known as the reparameterization trick. However, as the third and last step, we first instantiated the encoder. The procedure of creating the decoder was a little easier and only required two steps: declaring it and instantiating it.

##### Noise Addition

The simplest data augmentation method is adding noise to the EEG signal in time domain directly. This approach generates random Gaussian noise based on the statistical properties of the data. The mean of trials for the class for which trials were created was first calculated. Then, a Gaussian noise was generated with a mean of 0 and a standard deviation equal to the class mean. By merging randomly selected trials with generated noise, artificial frames were produced. The simple approach yielded trials with somewhat different numerical outcomes while maintaining the original properties of the waveform.

##### Hybrid Method

In the combined method, new data ws generated and added to the training dataset by using the methods of adding noise and VAE to the MCI data. The technique of adding noise is a preprocessing technique can be applied to train data before a model learns and makes inferences. The standard deviation values of 0.001 and the mean of zero were used to add noise to the data. Some other training data were also generated through the VAE method described in the previous sections. Additionally, the training dataset received the simultaneous additions of both types of newly generated data.

As test data, the classification of healthy individuals, MCI patients, and AD patients used 3 samples from the MCI category, 30 samples from the healthy category, and 17 samples from the AD group. In the 50 trials considered, several combinations of this test data were tried and tested.

## 3. Results

**Figure 2:**
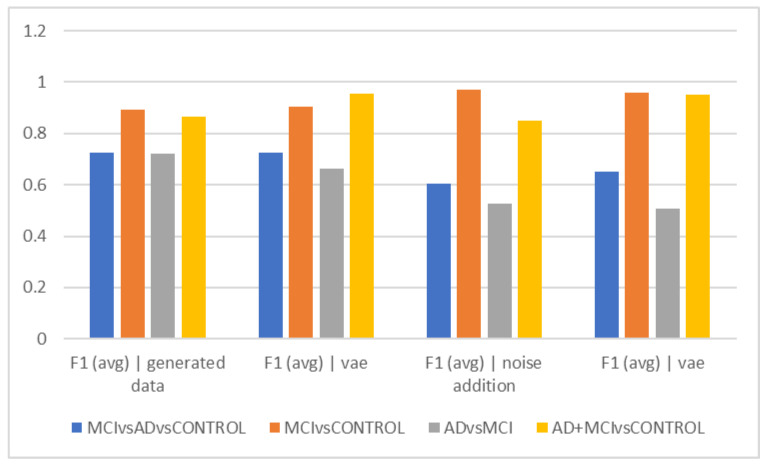
Comparison of results for a period of 5 s (accuracy).

Table 2 and Figure 2 show the average accuracy and F1Score criteria for the 50 trials for different time intervals of 5, 10, and 15 s when using data augmentation during training and when it was not employed. The classification of healthy and MCI samples is more accurately completed with the new data generated utilizing VAE and noise addition, according to the accuracy findings. Furthermore, it is evident from the F1Score results that the data augmented has an increased F1Score across all classifications (See Table 3, Table 4, Table 5, Table 6 and Table 7).

**Figure 3:**
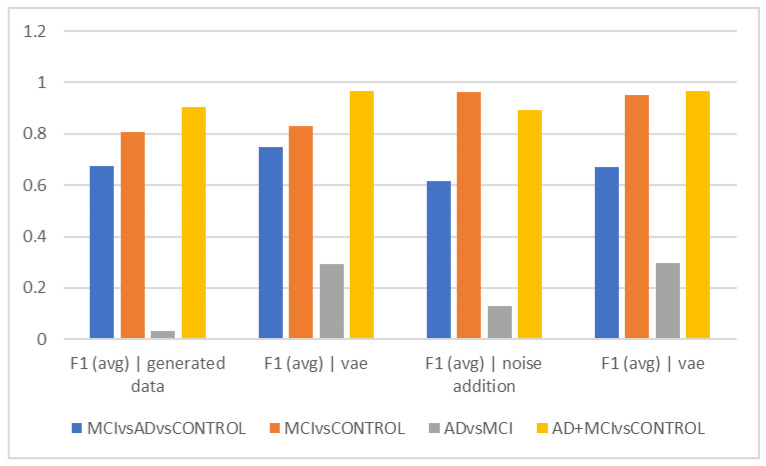
Comparison of results for 5 s (F1).

**Figure 4:**
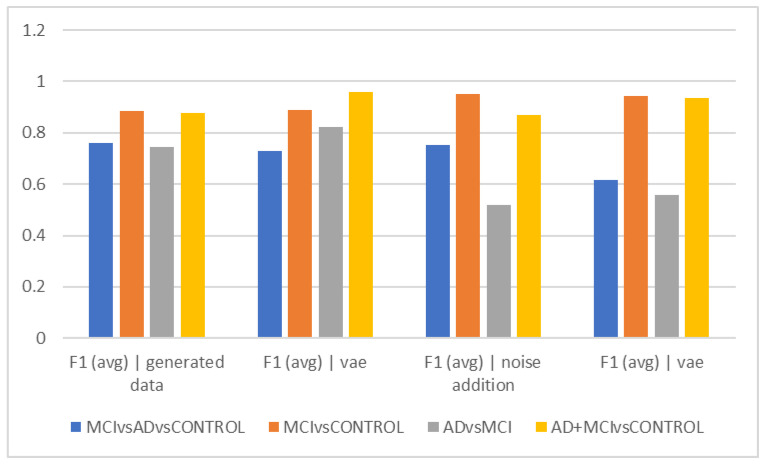
Comparison of results for a period of 10 s (accuracy).

**Figure 5:**
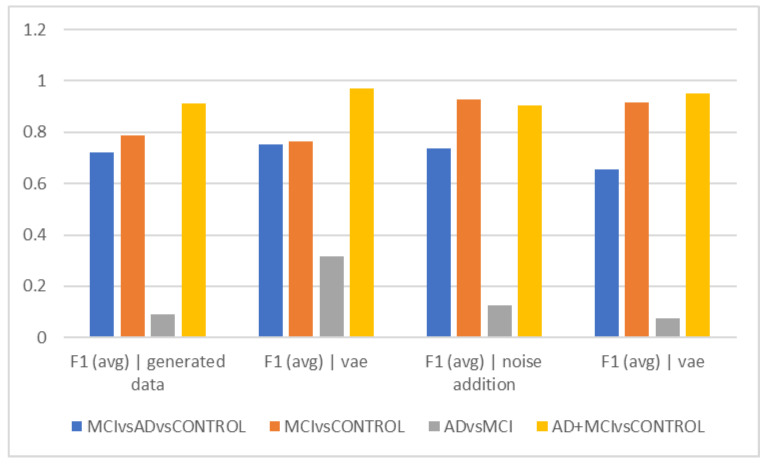
Comparison of results for a period of 10 s (F1).

**Figure 6:**
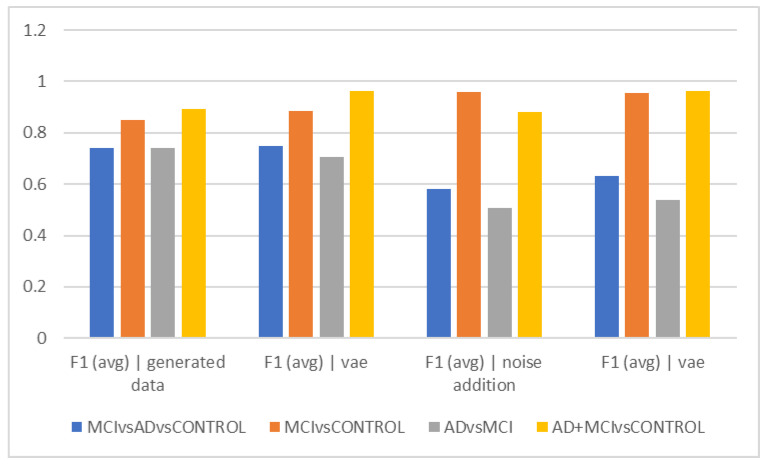
Comparison of results for a period of 15 s (accuracy).

**Figure 7:**
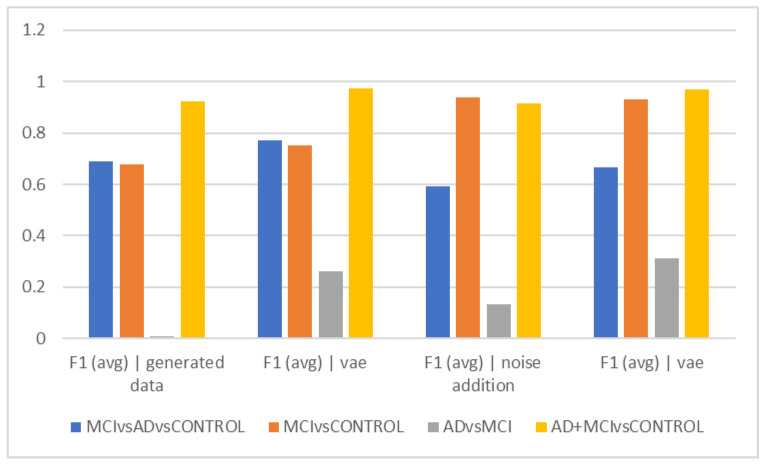
Comparison of results for a period of 15 s (F1).

As can be observed from the outcomes evaluated in all the different scenarios, the maximum accuracy gained from data augmentation methods is considerably higher than the raw data, illustrating the importance of data augmentation in Alzheimer’s data. On the other hand, the combined method demonstrated less efficiency than the other two of the three suggested methods, since none of the intervals of 5, 10, and 15 s considerably outperformed the other time intervals, which was the time each fared best in different classification methods. The noise-addition approach in both accuracy and F1Score metrics has the highest score when compared to the VAE in the two-class mode when the AD class is absent. The VAE method is most accurate when the AD class is present. Additionally, the suggested model is able to discriminate patient subjects (MCI and AD) from HC ones with considerable accuracy. The accuracy in MCI vs. HC mode is 97.2, while in AD + MCI vs. HC mode, it is 96.2. While the highest accuracy of the two augmented approaches did not differ significantly in the three-class mode, they are also less accurate in classifying classes than in the two-class mode (See Figure 2, Figure 3, Figure 4, Figure 5, Figure 6 and Figure 7).

## 4. Discussion

Analyzing EEG signals is a quick, inexpensive, and widely available method with a high temporal resolution that enables the research of the dynamic processes involved in the control of the intricate brain functioning system [23]. As a result, it can be used to detect cognitive impairment in the early stages of major neurocognitive disorders, such as Alzheimer’s disease and MCI [24]. In order to identify appropriate biomarkers for the early detection of severe neurocognitive disorders such as Alzheimer’s, the study of EEG signal data has therefore grown in importance in recent years [25]. Another important use of EEG signal data is the easier and more accurate differentiation of neurocognitive disorders from MCI. Due to the difficulty in differentiating between the two (AD and MCI) in the clinic and the similarities in the clinical picture, clinicians in this field, including psychiatrists and neurologists, are occasionally unable to distinguish between them accurately using only an interview and clinical and cognitive evaluation [26]. Techniques that can aid clinicians in the diagnosis and assist them in making better clinical decision assessments seem to be vital.

According to clinicians in this field, the clinical significance of this distinction and the more precise diagnosis of the two is that the treatment approaches for these two disorders are dissimilar. On the other hand, beginning treatment for Alzheimer’s disease as soon as possible helps to better control the disease and slow the progression of cognitive and behavioral symptoms. It can be a challenge to distinguish between persons who are entirely healthy and have cognitive complaints and those who have MCI since cognitive symptoms can often overlap in patients with different stages of the disease and are on a continuous spectrum.

To improve the accuracy of this distinction as much as possible, a variety of machine learning techniques have been applied in recent studies. Therefore, any technique that aids clinicians in making a more precise clinical diagnosis in this field will have clinical benefits. As a result, the current study’s goal was to apply various machine learning approaches to analyze and interpret the data generated from EEG signals in order to distinguish between Alzheimer’s disease, MCI, and the healthy population. Accuracy and F1scores were the two metrics employed in this study, and the results were interpreted based on these two parameters at intervals of 5, 10, and 15 s. As previously mentioned, three different data augmentation techniques were used in the field of machine learning to evaluate and interpret the data. Finally, they were compared in terms of accuracy in differentiating the three groups. This is due to the obvious difference in the amount of data in the different groups.

As shown in Figure 2a, using the noise-addition technique, the HC and MCI could be distinguished with the highest degree of accuracy (acc = 97.22%), while the Alzheimer’s group and MCI could be distinguished with the lowest level of accuracy (acc = 50.68%) using the hybrid technique. Therefore, it seems that the clinical challenge for therapists in differentiating between these two groups also exists in the interpretation of EEG findings. When interpreting the findings, it may be claimed that it appears that the MCI group’s attributes are so distinct from those of healthy individuals that their differentiation is carried out as precisely as possible. However, it has been difficult to distinguish MCI patients from Alzheimer’s patients due to the similarities in their characteristics and EEG findings. In other words, the EEG alterations in these people are more similar to those in Alzheimer’s patients and clearly different from the group of healthy people. The spectrum model of cognitive impairment, which sees MCI as a transitional state between healthy people and those with Alzheimer’s disease, may need to be updated in light of this. This finding can prove that about half of the patients with MCI also receive a diagnosis of Alzheimer’s disease within a period of 4 years [14,16,27,28]. On the other hand, this issue might be caused by the type of samples used in the dataset, as there might be individuals with more severe MCI who are more similar to individuals with milder types of Alzheimer’s disease in terms of features.

The best accuracy (72.08%) in separating the Alzheimer’s group from MCI was obtained when no data augmentation technique was applied, which can suggest that the data augmentation techniques utilized by machine learning are unable to differentiate between these two groups. The F1Score was used to compare groups in 5-s epochs, and in fact, we also took the data’s distribution into account (Figure 2d). The ability to distinguish the Alzheimer’s group from MCI was less than in Figure 2a, despite the general trend of the F1Score in separating groups being similar to Figure 2a. According to its interpretation, the accuracy of differentiating these two groups decreased when the heterogeneity of the groups and data was taken into consideration using the F1Score. According to F1Score, the biggest problem was in differentiating the Alzheimer’s group from MCI, and when VAEs and hybrid techniques were used, higher scores were obtained in this area.

Figure 2b shows that the graph’s overall trend in epochs of 10 s is comparable to Figure 2a. This indicates that the differentiation of the Alzheimer’s and MCI groups still has the lowest accuracy, and the VAE method worked with higher accuracy in this differentiation. The highest accuracy of differentiation is in differentiating healthy people from the total of Alzheimer’s and MCI people, and the VAEs method still performed the best. The interpretation of the above finding can again points to the greater similarity of the two groups of Alzheimer’s and MCI in the EEG findings and the more obvious difference with healthy people.

The VAE method is more effective than other ways in differentiating AD and MCI, according to the interpretation of Figure 2e, which looks at the F1Score at ten-second intervals. However, it is estimated to be less than the accuracy because of the data imbalance in this F1Score difference. Figure 2c demonstrates that similar to the previous figures, the accuracy trend in 15-s epochs is similar, with the maximum accuracy in differentiating between the normal and MCI groups as well as between healthy individuals and the sum of the other two groups. Still, distinguishing between those with Alzheimer’s disease and those with MCI remains the most difficult and least accurate task; in these cases, approaches that did not require more data and then the VAE method performed better. Based on the F1Score, hybrid and noise-addition techniques performed better in Figure 2f in differentiating between healthy individuals and MCI. Additionally, the hybrid approach had better effectiveness in separating MCI from Alzheimer’s disease.

It is clear from a thorough examination of all the results that using data augmentation techniques often improves the accuracy of distinguishing between groups. Hence, using these techniques is advised in future research. On the other hand, no appreciable differences between time intervals were found that would affect how well different data augmentation techniques differentiate between classes. The hybrid technique shows low accuracy in almost all trials when separating MCI from Alzheimer’s. One of the potential causes of the low accuracy of the model is that there are not many actual MCI patients in the data set and the model has not been trained on a wide range of MCI patients. Alzheimer’s patients are described as having moderate severity in the dataset’s description, which suggests that they may function similarly to and closely resemble people with more severe MCI. As a result of the variety of MCI patients, it is suggested to incorporate as many cases of this type as possible in the dataset. By doing so, the effect of this variety on the stages of the MCI samples can be reduced, and the model can more accurately distinguish between MCI and Alzheimer’s disease by taking into account MCI cases with varying severity. Based on our current knowledge in the field of classical machine learning projects that have used feature extraction (pca) and classical machine learning classifiers (svm), there is no similar study that has used data augmentation methods to improve accuracy. Therefore, it is not possible to compare the results of our study with previous works in this field.

The biggest difficulty in this study is differentiating MCI from Alzheimer’s. On the other hand, it is a challenge for clinicians to differentiate between the two. The VAE method has typically been able to achieve this distinction more effectively than alternative techniques. Therefore, the method of increasing data based on VAEs may be introduced as an effective parameter in increasing the accuracy of differentiating MCI from Alzheimer’s. The ability to distinguish between individuals with minor cognitive issues such as MCI, more serious ones such as Alzheimer’s, and healthy people is another benefit of adopting these techniques. The significance of this distinction for therapists is to prevent classifying healthy people as sick while also ensuring that those who have cognitive issues of any degree are identified quickly and are given the appropriate treatment. Therefore, the effectiveness of these approaches to distinguish between individuals with MCI and Alzheimer’s disease and the general population was also examined in this study. The VAE approach has been more successful in this differentiation when looking broadly at several time frames. Therefore, this approach is suggested when attempting to separate healthy individuals from those who have cognitive issues of any kind. Due to the lack of serious cognitive symptoms in the early stages of MCI, it may be challenging for clinicians to distinguish patients with MCI from healthy individuals. Noise addition and hybrid methods have consistently outperformed other ways of separating a group of healthy individuals from MCI, and in difficult cases in this field, these two strategies appear to be more effective than others. The originality of this work is that it used data augmentation techniques to improve classification accuracy, which have not been used in previous classical machine learning studies that use PCA feature extraction and traditional SVM machine learning classifiers. Approaches such as GAN and VAE have been used to augment synthetic EEG data in methods that use deep learning models to distinguish between classes.

One of the study’s limitations is the imbalanced dataset and lack of variation, particularly in the MCI group, where the abundance of features causes an imbalanced distribution of data for analysis. Future research should take into account the fact that disease symptoms, etiology, and kind of treatment might vary widely, which may have had an impact on the interpreted EEG data. Additionally, disease symptoms may change with age, which leads to changes in EEG features. It is proposed that more emphasis be given to methods that address this issue in future research, given that the primary challenge is to reliably distinguish the Alzheimer’s group from MCI (which, of course, is a challenge for therapists in the clinic as well). This issue can be resolved by collecting datasets more frequently and with a wider variety, as well as by using machine learning techniques for data augmentation (such as the VAE method this paper proposes).

## 5. Conclusions

In this study, three classes—AD, MCI, and HC—were successfully distinguished using EEG data. The accuracy attained in the classification of three classes after data augmentation using the VAE model increased by 3% in comparison to the pre-augmentation stage. This shows how useful EEG data augmentation techniques are. In future works, we have decided to use generative adversarial networks (GAN) and variational auto encoder networks (VAE) to generate artificial EEG data due to the limited number of available EEG data samples. We will also use a convolutional neural network (CNN) model based on transfer learning to learn distinctive features from raw EEG signals.

## Figures and Tables

**Table 1:** Features extracted from electroencephalographic signals.

Feature Type	Channels	Feature	Number of Features
Average power spectrum density	FP1, FP2, F7, F3, Fz, F4, F8, T7, C3, Cz, C4, T8, P7, P3, Pz, P4, P8, O1, O2	Average power spectrum density in the delta, theta, alpha, beta and gamma frequency bands for 19 channels	**95**
Statistical features	FP1, FP2, F7, F3, Fz, F4, F8, T7, C3, Cz, C4, T8, P7, P3, Pz, P4, P8, O1, O2	Mean, variance, and ZCR for 19 channels	**57**

**Table 2:** Average accuracy and F1Score criteria for 50 trials for different time intervals. Results for 5 s (accuracy).

	F1 (avg)|Generated Data	F1 (avg)|Vae Generated Data	F1 (avg)|Noise Addition	F1 (avg)|Vae Generated Data|Noise Addition
MCI vs. AD vs. CONTROL	0.7238	0.7256	0.6064	0.6494
MCI vs. CONTROL	0.8936	0.9058	0.9722	0.96
Ad vs. MCI	0.7208	0.6646	0.5256	0.5068
AD+MCI vs. CONTROL	0.866	0.9534	0.8508	0.9524

**Table 3:** Average accuracy and F1Score criteria for 50 trials for different time intervals. Results for a period of 5 s (F1).

	F1 (avg)|Generated Data	F1 (avg)|Vae Generated Data	F1 (avg)|Noise Addition	F1 (avg)|Vae Generated Data|Noise Addition
MCI vs. AD vs. CONTROL	0.6765	0.75	0.6154	0.6692
MCI vs. CONTROL	0.8089	0.8292	0.9618	0.95
Ad vs. MCI	0.031	0.2949	0.1289	0.2978
AD+MCI vs. CONTROL	0.9034	0.9652	0.892	0.9658

**Table 4:** Average accuracy and F1Score criteria for 50 trials for different time intervals. Results for 10 s (accuracy).

	F1 (Avg)|Generated Data	F1 (avg)|Vae Generated Data	F1 (avg)|Noise Addition	F1 (avg)|Vae Generated Data|Noise Addition
MCI vs. AD vs. CONTROL	0.7605	0.7282	0.7518	0.6162
MCI vs. CONTROL	0.8844	0.8886	0.9512	0.9426
AD vs. MCI	0.7434	0.821	0.5202	0.5582
AD+MCI vs. CONTROL	0.8768	0.9592	0.871	0.9342

**Table 5:** Average accuracy and F1Score criteria for 50 trials for different time intervals. Results for 10 s (F1).

	F1 (avg)|Generated_Data	F1 (avg)|Vae Generated_Data	F1 (avg)|Noise_Addition	F1 (avg)|Vae Generated_Data|Noise_Addition
MCI vs. AD vs. CONTROL	0.7208	0.7532	0.7382	0.6534
MCI vs. CONTROL	0.7888	0.765	0.9278	0.9178
AD vs. MCI	0.091	0.3182	0.1239	0.0753
AD+MCI vs. CONTROL	0.9103	0.971	0.9062	0.9526

**Table 6:** Average accuracy and F1Score criteria for 50 trials for different time intervals. Results for 15 s (accuracy).

	F1 (avg)|Generated_Data	F1 (avg)|Vae Generated_Data	F1 (avg)|Noise_Addition	F1 (avg)|Vae Generated_Data|Noise_Addition
MCI vs. AD vs. CONTROL	0.7408	0.7486	0.5818	0.6312
MCI vs. CONTROL	0.8488	0.8832	0.9572	0.9534
AD vs. MCI	0.741	0.7044	0.506	0.5366
AD+MCI vs. CONTROL	0.8917	0.9622	0.8802	0.9622

**Table 7:** Average accuracy and F1Score criteria for 50 trials for different time intervals. Results for 15 s (F1).

	F1 (avg)|Generated_Data	F1 (avg)|Vae Generated_Data	F1 (avg)|Noise_Addition	F1 (avg)|Vae Generated_Data|Noise_Addition
MCI vs. AD vs. CONTROL	0.6885	0.7714	0.5908	0.6656
MCI vs. CONTROL	0.6769	0.7524	0.937	0.9318
AD vs. MCI	0.01	0.2622	0.1333	0.3108
AD+MCI vs. CONTROL	0.9224	0.972	0.9154	0.9718

## Data Availability

Not applicable.
